# The Effectiveness of Oral Nutrition Supplementation on Growth Indicators, Dietary Intake, Appetite, and Health Outcomes Among Underweight Preschool Children in Urban Area of Kuala Lumpur, Malaysia: A Multi-Centre Community-Based Randomised Controlled Trial

**DOI:** 10.3390/nu18162714

**Published:** 2026-08-19

**Authors:** Abdul Hakim Abdul Wahid, Nur Hana Hamzaid, Haslina Abdul Hamid, Rosnah Sutan, Zahara Abdul Manaf

**Affiliations:** 1 Centre for Healthy Ageing & Wellness (H-Care), Faculty of Health Sciences, Universiti Kebangsaan Malaysia, Kuala Lumpur 50300, Malaysia; p154146@siswa.ukm.edu.my; 2 Centre for Rehabilitation & Special Needs Studies (ICaReHab), Faculty of Health Sciences, Universiti Kebangsaan Malaysia, Kuala Lumpur 50300, Malaysia; 3 Centre for Community Health Studies (ReaCH), Faculty of Health Sciences, Universiti Kebangsaan Malaysia, Kuala Lumpur 50300, Malaysia; 4 Department of Public Health Medicine, Faculty of Medicine, Universiti Kebangsaan Malaysia, Kuala Lumpur 50600, Malaysia

**Keywords:** oral nutritional supplementation, childhood undernutrition, preschool children, weight-for-age z-score, catch-up growth, dietary adequacy, appetite, randomised controlled trial, Malaysia

## Abstract

**Background/Objectives**: Undernutrition remains a public health concern among preschool children in Malaysia. Although oral nutritional supplementation (ONS) globally has been recommended to improve nutritional status, evidence from community-based randomised controlled trials among underweight Malaysian preschool children remains limited. This study evaluated the effectiveness of ONS on growth, dietary intake, appetite, and common illnesses among underweight preschool children in Kuala Lumpur, the capital city of Malaysia. **Methods**: A multi-centre, community-based, cluster-randomised controlled trial was conducted among underweight preschool children aged 4–6 years from 16 government-subsidised preschools. Participants were assigned to receive ONS plus nutrition education (ONS + NE) or nutrition education alone (NE only) for 90 days. Anthropometric measurements, dietary intake, appetite, attainment of catch-up growth, and parent-reported common illnesses were assessed. Between-group differences were analysed using analysis of covariance adjusted for baseline values. **Results**: Ninety-six underweight preschool children were randomised to ONS plus nutrition education (ONS + NE, *n* = 56) or nutrition education alone (NE only, *n* = 40). Compared with the NE group, children receiving ONS + NE demonstrated significantly greater improvements in weight-for-age z-scores at day 60 and day 90 (*p* < 0.05). The ONS + NE group also achieved significantly higher catch-up growth attainment at day 60 (116.6 ± 38.5% vs. 99.1 ± 17.4%; *p* = 0.029) and day 90 (114.6 ± 37.2% vs. 95.5 ± 23.8%; *p* = 0.021). In addition, significantly higher energy, macronutrients, selected micronutrient intakes, and appetite scores were observed in the ONS + NE group (all *p* < 0.05). **Conclusions**: ONS combined with nutrition education improved weight-related growth, dietary adequacy, catch-up growth energy achievement, and appetite among underweight preschool children. ONS may serve as an effective community-based strategy for nutritional rehabilitation in this population. Trial registration: ISRCTN15093638.

## 1. Introduction

Undernutrition among children under five years of age remains a major global public health concern, particularly in low- and middle-income countries. According to the World Health Organisation (WHO), 148 million children under five were stunted, and 45 million were wasted [1]. Children experiencing underweight, stunting, and wasting face increased morbidity, impaired cognitive development, and long-term adverse health consequences [2,3]. In the Asian region, including in Association of Southeast Asian Nations (ASEAN) countries, childhood undernutrition persists despite rapid urbanisation and economic development [4,5]. Currently, Malaysia is an upper-middle-income country that is simultaneously facing the triple burden of malnutrition, where undernutrition, overnutrition, and micronutrient deficiencies coexist within the population [6]. The National Health Morbidity Survey for Children and Maternal in 2022 has shown that underweight among children remains a persistent undernutrition issue in Malaysia (15.3%) and Kuala Lumpur (12.4%) [7]. A recent study conducted in Kuala Lumpur highlighted the prevalence of undernutrition among preschoolers in poor urban households, where 34.7% of the preschoolers in this study experienced at least one form of undernutrition [8]. These poor urban households in Kuala Lumpur may experience limited access to nutritious foods, unhealthy feeding practices, recurrent infections, and inadequate dietary quality, all of which may negatively affect children’s growth and overall health outcomes [9].

Adequate dietary intake during early childhood is essential to support optimal physical growth, immune function, and cognitive development [10]. Preschool years represent a critical window for catch-up growth, where insufficient energy and nutrient intake may lead to growth faltering and increased susceptibility to illness. Previous studies have demonstrated that underweight children often fail to meet recommended dietary requirements for energy, protein, and micronutrients, consequently affecting anthropometric indicators such as weight-for-age, height-for-age, and body mass index-for-age [9,10,11]. Poor nutritional intake may also impair appetite regulation by disrupting normal hunger and satiety signalling, reducing children’s willingness to consume balanced meals and further exacerbating nutritional deficiencies [12,13]. Inadequate nutrition during this developmental stage has additionally been associated with reduced physical activity, impaired learning capacity, and frequent common illnesses, highlighting the importance of early nutritional intervention among underweight preschool children [3,14,15].

Oral nutrition supplementation (ONS) has been increasingly recognised as an effective nutritional intervention for children who are unable to meet their nutritional requirements through habitual dietary intake alone. ONS products are formulated to provide high energy, complete essential protein, and essential micronutrients that support catch-up growth and overall nutritional rehabilitation among undernourished children [16,17]. Previous studies have demonstrated that ONS may contribute to improved body weight, height, body mass index, growth indicators, and micronutrient status among children with growth faltering or at nutritional risk [17,18,19]. In addition to anthropometric improvements, ONS has also been associated with enhanced appetite [20] and improved dietary adequacy [16,19] when administered consistently alongside dietary counselling or nutrition education. However, the majority of the studies were conducted in clinical or hospital settings that may only reflect children in clinical conditions, while studies conducted in community settings remain limited and needed. Additionally, despite growing evidence internationally, the effectiveness of ONS may vary according to population characteristics, baseline nutritional status, duration of supplementation, adherence, and socio-environmental factors, highlighting the need for context-specific evaluation among Malaysian preschool children. Notably, a recent Malaysian community-based intervention study [19] reported that daily intake of nutrient-dense ONS for 180 days significantly improved height-for-age z-scores, weight-for-age z-scores, and micronutrient intake among stunted and at-risk children aged 1 to 3 years. However, this study employed a single-arm intervention design without a control group, included younger children, was conducted in a rural area of northeast of Malaysia, and mainly focused on linear growth outcomes and nutrient adequacy. Therefore, evidence from controlled community-based trials evaluating ONS among undernourished preschool children (4–6 years) residing in urban low-income communities, while simultaneously assessing growth, dietary intake, appetite, and catch-up growth, remains limited.

Nevertheless, evidence regarding the effectiveness of ONS among underweight preschool children in Malaysian urban community settings remains limited. Furthermore, no published study on a multi-centre randomised controlled trial with two study arms has evaluated the effectiveness of ONS among underweight preschool children in Kuala Lumpur, the capital city of Malaysia. Kuala Lumpur was chosen because, despite being the most urbanised city in the country, undernutrition persists among children from low-income households, reflecting the coexistence of urban poverty, food insecurity, and nutritional inequalities. Given the persistent prevalence of underweight among Malaysian children and the multifactorial nature of undernutrition involving dietary inadequacy, poor appetite, and recurrent illnesses, there is a need for a comprehensive community-based intervention approach. Therefore, this study aimed to determine the effectiveness of oral nutrition supplementation on growth indicators, dietary intake, appetite, and common illnesses among underweight preschool children in urban areas of Kuala Lumpur through a multi-centre community-based randomised controlled trial.

## 2. Materials and Methods

This study was a multi-centre, community-based, prospective, parallel-group randomised controlled trial conducted to evaluate the effectiveness of oral nutritional supplementation (ONS) combined with nutrition education on growth indicators, dietary intake, appetite, and health outcomes among underweight preschool children in Kuala Lumpur, Malaysia.

### 2.1. Eligibility Criteria

Participants included in this study were underweight preschool children recruited from selected government-funded preschools of the Community Development Department (KEMAS) around Kuala Lumpur, Malaysia. Eligible participants fulfilled the following inclusion criteria: (i) Malaysian citizen; (ii) boys and girls aged 4–6 years; and (iii) weight-for-age z-score (WAZ) between −3 and <−2 standard deviations (SD) based on the World Health Organisation (WHO) Child Growth Standards and WHO Growth Reference 2007 [21,22].

Children were excluded if they either had severe malnutrition requiring hospital intervention, were immunocompromised, had undergone surgery within three months prior to recruitment, had serious infections or injuries, congenital diseases or malformations, chronic diseases, or were receiving medications that could potentially interfere with growth outcomes. Children with lactose intolerance, hypersensitivity to the study product ingredients, aversion to milk consumption, or who had received nutritional or medical interventions targeting growth within the preceding six months were also excluded.

### 2.2. Ethical Procedures

The study protocol received ethical approval from the Research Ethics Committee of Universiti Kebangsaan Malaysia (RECUKM) (Reference No: JEP-2024-778). Approval to conduct this study at selected preschools was obtained from the Community Development Department (KEMAS), Ministry of Rural and Regional Development, Malaysia. The study protocol was registered under the International Standard Randomised Controlled Trial Number (ISRCTN15093638; date of registration 18 Mar 2026). The study was conducted in accordance with the ethical principles of the Declaration of Helsinki and adhered to the Consolidated Standards of Reporting Trials (CONSORT) guidelines for the design, conduct, and reporting of randomised controlled trials. Written informed consent was obtained from parents or legal guardians prior to participation. Participants’ caregivers were informed regarding study objectives, procedures, risks, and potential benefits. Confidentiality and anonymity of participants were maintained throughout the study, and all data were accessible only to authorised research personnel through password-protected institutional databases.

### 2.3. Sampling, Recruitment, and Randomisation

This study employed a multi-centre, community-based, prospective, parallel-group randomised controlled trial design using cluster randomisation at the preschool level. Cluster randomisation was selected to minimise intervention spillover and potential crossover between participants, as children within the same preschool environment may share meals, activities, and behavioural influences that could affect study outcomes.

A list of government-subsidised preschools within Kuala Lumpur was obtained from the relevant preschool administrative authorities and organised according to the seven administrative sub-districts of Kuala Lumpur. A total of 120 preschools were initially identified across the seven sub-districts and served as the source population for recruitment. Invitation letters and study information were distributed to all identified preschools. Among these, 95 preschools responded to the invitation. Among the responding preschools, 36 agreed to participate and fulfilled the study requirements, including willingness to participate throughout the intervention period, availability of eligible preschool children, and administrative approval, forming the final sampling frame.

From these 36 eligible preschools, 16 centres were randomly selected using a computer-generated simple random selection procedure while ensuring representation across the seven administrative sub-districts. Following centre selection, cluster randomisation was conducted at the preschool level using a computer-generated random allocation sequence. The selected preschools were randomly assigned in a 1:1 ratio to either the intervention group receiving oral nutritional supplementation combined with nutrition education (ONS + NE) or the control group receiving nutrition education only (NE), resulting in eight preschools allocated to each study arm.

**Figure 1:**
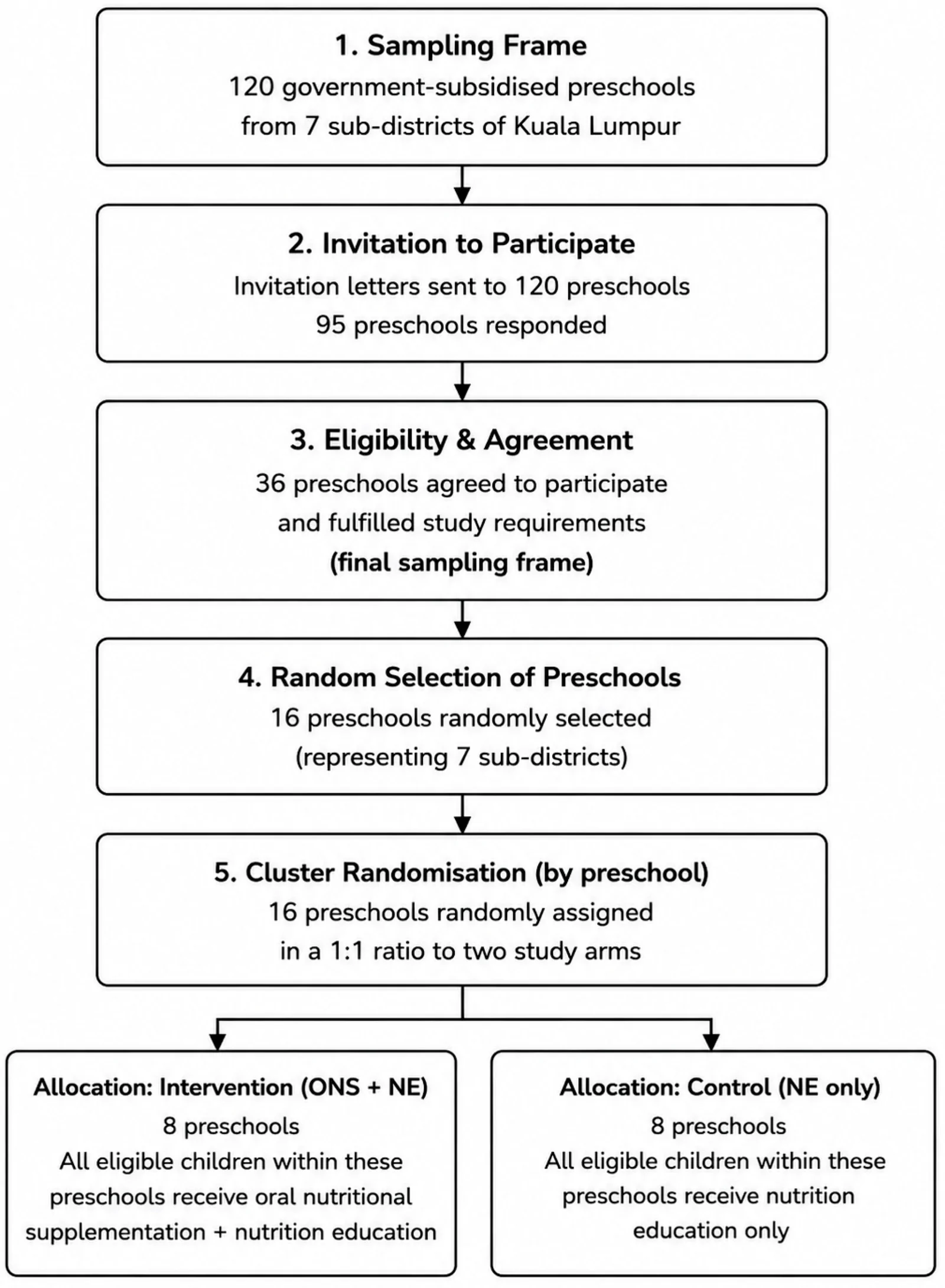
Flow diagram of the preschool sampling, recruitment, selection, and cluster randomisation process for the multi-centre community-based randomised controlled trial.

Following allocation, all children within each selected preschool were screened for eligibility according to the predefined inclusion and exclusion criteria. Parents or legal guardians of eligible children were approached and provided with detailed information regarding study procedures before obtaining written informed consent. Children from the same preschool received the same intervention allocation throughout the study period to minimise intervention spillover and potential crossover. The overall sampling, recruitment, and cluster randomisation process is illustrated in Figure 1.

### 2.4. Sample Size Calculation

Sample size estimation was performed using G*Power version 3.1 based on repeated-measures mixed analysis of variance (ANOVA) to detect differences between intervention groups across study time points. The calculation assumed a medium effect size (f = 0.25) following Cohen’s guideline [23], a significance level of 0.05, statistical power of 80%, two study groups, and four repeated measurements. The minimum calculated sample size per arm was estimated at 24 participants. After accounting for a 20% drop-out rate based on previous ONS intervention studies [16], the final sample size required was 58 participants, corresponding to 29 participants per study arm.

### 2.5. Data Collection Procedures

#### 2.5.1. Anthropometric Measurements

Anthropometric assessments were conducted at baseline, day 30, day 60, and day 90 by trained researchers using standardised procedures. Body weight was measured to the nearest 0.1 kg using a calibrated digital weighing scale (Seca 803, Hamburg, Germany), while height was measured to the nearest 0.1 cm using a portable stadiometer (Seca 216, Hamburg, Germany). Participants were measured barefoot and wearing light clothing. The weighing scale was calibrated against standard reference weights at each measurement session to ensure measurement accuracy.

Body mass index (BMI) was calculated as body weight divided by height squared (kg/m^2^). Measurements were performed in duplicate, and a third measurement was conducted when the difference between readings exceeded 10%. Anthropometric z-scores including weight-for-age (WAZ), height-for-age (HAZ), and BMI-for-age (BAZ) were generated using WHO Anthro software (version 3.2.2) [24] and WHO AnthroPlus Software (version 1.0.4) [25] according to WHO Child Growth Standards and WHO Growth Reference (2007) for children aged 5–19 years [21,22]. Nutritional status classifications were based on WHO reference criteria. Underweight status was defined as WAZ between −3 SD and <−2 SD, stunting was defined as HAZ between −3 SD and <−2 SD, and wasting was defined as BAZ between −3 SD and <−2 SD [21,22]. All anthropometric equipment used throughout the study underwent routine calibration according to manufacturer recommendations and standard measurement protocols.

#### 2.5.2. Dietary Assessment

Dietary intake was assessed using a quantitative three-day 24 h dietary recall consisting of two weekdays and one weekend day at baseline (day 0), day 60, and day 90. The recalls included all foods, beverages, and ONS consumed during the assessment period to estimate energy and nutrient intake. Diet recalls were conducted via telephone interviews with parents or the primary caregivers. Interviews were conducted by trained research assistants under the supervision of a nutritionist using a standardised probing guide to improve recall of foods and beverages during dietary reporting. The researcher provided a structured food and fluid intake log for teachers to record children’s dietary intake during school hours. The teachers were trained by researchers to record the food and fluid intake of the children during school hours.

Household measurement tools and food photographs from the children and toddlers’ food atlas [26] were used to assist portion size estimation during the telephone interviews. Parents and caregivers were provided with soft copies of the household measurement booklet and food album for reference during dietary assessment. Nutrient analysis and total daily energy intake calculations were performed using Nutritionist Pro™ Diet Analysis software (version 5.1.0; Axxya Systems, Redmond, WA, USA). Recipes unavailable in the database were manually entered using standard recipes and ingredient quantities. Food composition data were primarily obtained from the Nutrient Composition of Malaysian Foods and supplemented by the Malaysian Food Composition Database (MyFCD) [27,28] and food product labels where necessary.

To improve dietary data quality, the plausibility of reported energy intake was assessed according to the method proposed by Black and Cole (2000) [29] through calculation of the ratio between reported energy intake (EI) and estimated energy expenditure (EE). Estimated EE was calculated by multiplying the basal metabolic rate (BMR), derived using the Schofield equation, by a physical activity level (PAL) of 1.60, assuming moderate physical activity among preschool children. Plausibility limits were determined using a 99% confidence interval based on within-subject coefficients of variation of 23% for energy intake and 8.2% for energy expenditure. Based on this approach, an acceptable EI/EE ratio of 0.30–1.70 was applied, with participants falling outside this range classified as implausible reporters and excluded from dietary intake analyses. A total of three over-reporters were excluded, resulting in a final sample of 61 participants for dietary analyses.

#### 2.5.3. Catch-Up Growth Assessment

Catch-up growth energy requirements were estimated using an adapted Peterson formula [30] to determine the energy needed to support accelerated growth among underweight children. The estimated catch-up growth energy requirement was calculated as follows:

(1) $$Catch\text{-}up\;\;Growth\;\;Energy\;\;Requirement\;(kcal/day)=\frac{RNI\;\;Energy\;\;for\;\;Weight\;\;Age\;\;\left(kcal/kg\right)\;\times \;IBW\;\;\left(kg\right)}{Actual\;\;Body\;\;Weight\;\;\left(kg\right)}$$

where weight age refers to the age at which the child’s current body weight corresponds to the 50th percentile of the WHO growth reference, and ideal body weight (IBW) refers to the body weight at the 50th percentile for the child’s chronological age. Recommended nutrient intake (RNI) values were obtained from the Malaysian Recommended Nutrient Intakes (RNI) 2017. The percentage of catch-up growth achieved was subsequently calculated by comparing the child’s observed daily energy intake with the estimated catch-up growth energy requirement and multiplying by 100. Values ≥100% indicated achievement of the estimated catch-up growth energy requirement.

#### 2.5.4. Appetite and Common Illness Assessments

Children’s appetite was assessed at baseline and day 90 using a parent-reported visual analogue scale adapted from a previously published study [17]. Parents were asked to rate their child’s overall appetite in the preceding period on a scale ranging from 0 to 5, where 0 represented very poor appetite, and 5 represented very good appetite. Higher scores indicated a better appetite. The assessment was completed by parents or primary caregivers based on their observations of the child’s eating behaviour and food intake during the preceding week.

Common illness outcomes were assessed at baseline and day 90 using a parent-reported checklist adapted from previous studies by Vernacchio et al. [31] and Khadilkar et al. [20,32]. Parents or primary caregivers were asked to report on the occurrence of common childhood illnesses experienced by their child, including fever, cough, flu, diarrhoea, vomiting, and other minor illnesses. Throughout the intervention period, parents were instructed to observe their child’s health status and illness episodes; information was collected retrospectively during the assessment of visits based on parental observations.

### 2.6. Intervention

Participants in the intervention arm received oral nutritional supplementation together with nutrition education, whereas participants in the control arm received nutrition education alone. Nutrition education was delivered by registered nutritionists and dietitians using standardised educational materials adapted from Malaysia’s Ministry of Health guidelines [33,34,35]. Educational content included parenting feeding practices, balanced dietary intake, food variety, dietary quality improvement, and strategies to improve children’s appetite. Education sessions included a half-day seminar and a cooking demonstration conducted during the baseline period.

Children in the intervention group received a commercially available oral nutritional supplement, Resurge Junior^®^ (Quantum Upstream, Kuala Lumpur, Malaysia), a nutritionally complete formula containing energy, protein, essential fatty acids, prebiotics, and micronutrients. Each serving consisted of one sachet (48 g powder) reconstituted with 170 mL of water, providing approximately 219 kcal of calories, 6.7 g protein, 28.3 g carbohydrate, and 7.3 g fat per serving/sachet. The macro- and micronutrient composition of the ONS is presented in Appendix A (Table A1).

### 2.7. ONS Delivery and Subject Compliance

Children in the intervention group received two sachets of ONS daily throughout the 90-day intervention period. One serving of ONS was prepared and administered by trained preschool teachers during school hours in the morning, while the second serving was provided to the children before returning home at noon. The 90 days of ONS supplementation were given only during school days, which excluded weekends and public and school holidays in Malaysia. This supplementation schedule was implemented to ensure adequate nutrient distribution throughout the day and to optimise adherence to the protocol. Before the study began, teachers underwent training on ONS preparation procedures, dosage administration, storage methods, and compliance recording. Daily ONS intake was recorded using a food and fluid intake log and verified through collection of used sachets during follow-up visits. According to a previous, similar study, participants who consumed at least 75% of the prescribed ONS servings throughout the intervention period were considered compliant with the intervention protocol [17]. ONS tolerance and acceptance were assessed on day 7 of the intervention.

### 2.8. Data Management and Statistical Analysis

Data quality assessment was performed in two stages. First, all data entered into Microsoft SharePoint were verified against questionnaires to ensure completeness and accuracy. Subsequently, verified data were transferred into IBM Statistical Package for Social Sciences (SPSS) Statistics version 30.0 (IBM Corp., Armonk, NY, USA) for data cleaning and statistical analysis. Data cleaning procedures included consistency checks, identification of missing values, and verification of implausible observations against source documents where necessary. Access to the study database was restricted to authorised research personnel through password-protected institutional platforms. Continuous variables were presented as the mean ± standard deviation (SD) for normally distributed data and the median with interquartile range (IQR) for non-normally distributed data, while categorical variables were presented as frequencies and percentages. Data normality was assessed using the Kolmogorov–Smirnov test.

As participants were randomised at the preschool level, the potential impact of clustering was assessed before the primary analyses. The intracluster correlation coefficient (ICC) for the primary outcome (baseline WAZ), estimated using a one-way random-effects analysis, was negligible (ICC < 0.001), indicating minimal between-preschool variation. Furthermore, one-way analysis of variance showed no significant differences in WAZ, HAZ, or BAZ between preschools at any assessment time point (all *p* > 0.05). Therefore, the effect of clustering on the outcome measures was considered minimal, and individual-level analyses were performed.

Baseline characteristics between intervention groups were compared using independent-sample *t*-tests for continuous variables and chi-square tests or Fisher’s exact tests for categorical variables, as appropriate. The primary outcomes, which are the anthropometric outcomes, were analysed according to intention-to-treat (ITT) analysis using the Baseline Observation Carried Forward (BOCF) approach to account for missing follow-up measurements. Changes in anthropometric outcomes, including body weight, height, BMI, WAZ, HAZ, and BAZ, were evaluated across study time points. The secondary outcomes, including dietary intake, appetite, common illness, and catch-up growth energy requirement analyses, were performed using complete-case data because these outcomes required complete dietary records and questionnaire responses, and missing values could not be reliably imputed. Dietary outcomes, including daily energy intake, macronutrients, micronutrients, and catch-up growth energy requirement percentages, were calculated using the mean values obtained from the three-day dietary recall assessments and a food and fluid log at school. Participants identified as implausible reporters based on EI/EE plausibility assessment were excluded from dietary analyses.

Between-group differences in anthropometric outcomes, catch-up growth, and appetite scores at follow-up assessments were analysed using analysis of covariance (ANCOVA), with baseline measurements included as covariates to adjust for initial differences between intervention groups. Adjusted mean differences with corresponding 95% confidence intervals were estimated where appropriate. Partial eta squared (partial η^2^) was reported to quantify the magnitude of the intervention effect. Effect sizes were interpreted according to Cohen’s recommendations, where 0.01, 0.06, and 0.14 represent small, medium, and large effects, respectively. Dietary intake variables were compared between groups using independent *t*-tests, while categorical variables of common illness outcomes were analysed using chi-square tests or Fisher’s exact tests, as appropriate. All statistical analyses were conducted using a two-sided significance level, and statistical significance was established at *p* < 0.05.

## 3. Results

**Figure 2:**
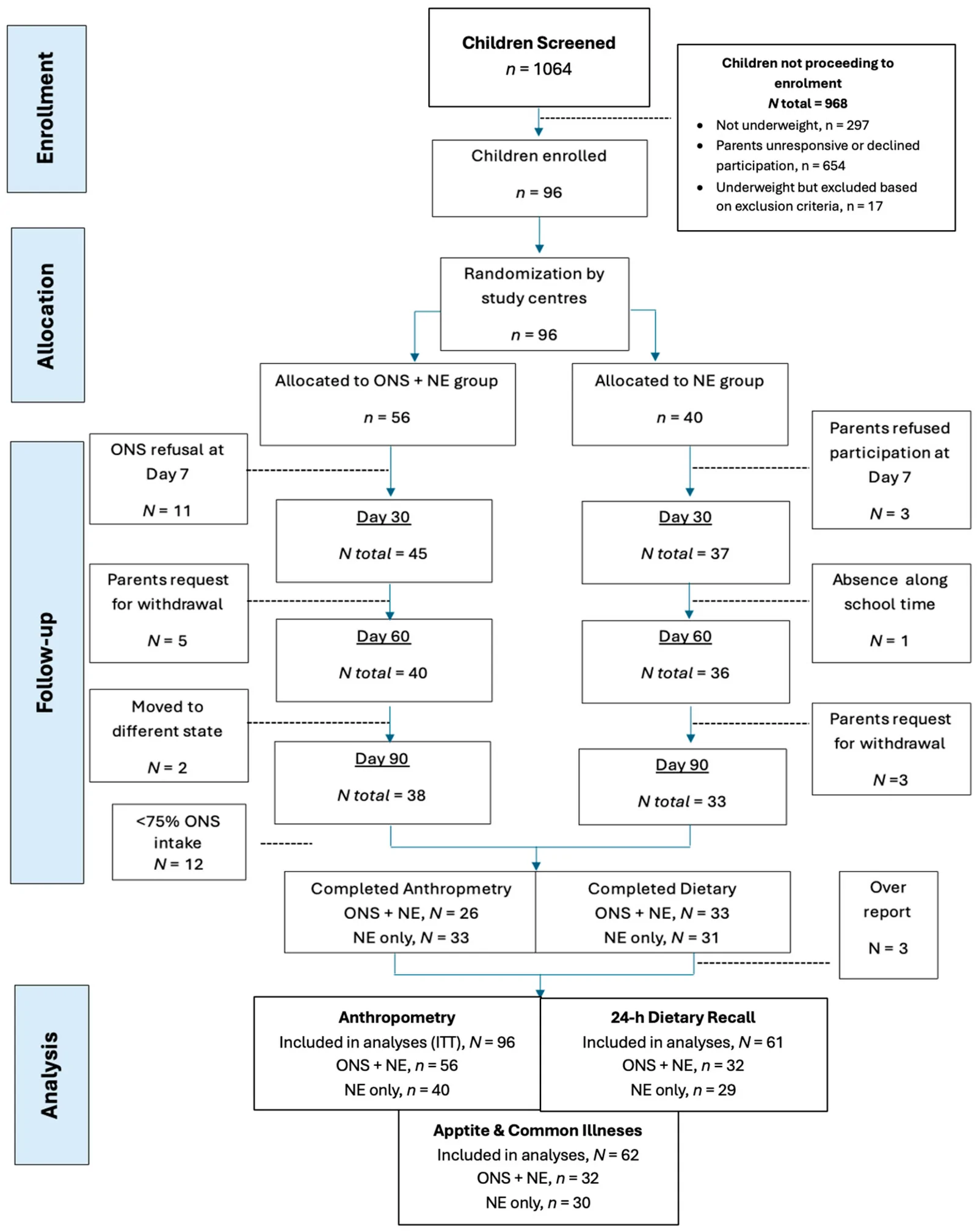
Study CONSORT flow chart.

A total of 1064 preschool children were screened for study participation. Following screening against the predefined inclusion and exclusion criteria, 96 eligible children were enrolled and randomised by study centres into the ONS + NE group (*n* = 56) and the nutrition NE group (*n* = 40). During follow-up, participant attrition occurred due to ONS refusal, parental withdrawal, relocation, school absence, and low ONS compliance (<75% intake). By day 90, 38 children in the ONS + NE group and 33 children in the NE-only group remained in the follow-up assessments. Completed anthropometric assessments were obtained from 26 children in the ONS + NE group and 33 children in the NE-only group, whereas completed dietary assessments were available for 33 and 31 children, respectively. Overall, 96 children were included in the anthropometric analyses, 61 in the dietary intake analyses, and 62 in the appetite and common illness analyses. The overall flow of participants through the study is presented in Figure 2.

### 3.1. Sociodemographic Characteristics and Intervention Adherence

Table 1 presents the baseline sociodemographic characteristics of the study participants. A total of 96 children were included, comprising 56 children in the oral nutritional supplementation with nutrition education (ONS + NE) group and 40 children in the nutrition-education-only (NE-only) group. The mean age of participants was 5.5 ± 0.6 years, and slightly more than half were male (54.2%). Most children were born full-term (92.7%). The majority of fathers and mothers had secondary education or below, and most mothers were not working. More than half of the fathers were smokers, whereas almost all mothers were non-smokers. Most households had monthly parental incomes below RM 2559 (USD 605.8). The majority of parents were married. No significant differences were observed between the ONS + NE and NE groups across all sociodemographic variables at baseline (all *p* > 0.05), indicating comparability between groups prior to intervention.

Intervention adherence among participants allocated to the ONS + NE group is summarised in Table 2. Of the 56 participants allocated to the intervention group, 11 (19.6%) refused ONS within the first 7 days of the intervention, while 38 (67.9%) completed the day 90 assessment. The mean percentage of prescribed ONS consumed throughout the intervention was 54.4 ± 31.2%. Based on the predefined compliance criterion of consuming at least 75% of the prescribed ONS (equivalent to an average intake of ≥1.5 servings/day), 26 participants (46.4%) were classified as compliant and were subsequently included in the per-protocol sensitivity analyses.

### 3.2. Primary Outcomes

#### 3.2.1. Baseline Anthropometrics

Table 3 presents the baseline anthropometric characteristics of the children enrolled in the study. A total of 96 children participated, comprising 56 children in the oral nutritional supplementation with nutrition education (ONS + NE) group and 40 children in the nutrition-education-only (NE-only) group. No significant differences were observed between groups in terms of age, sex distribution, body weight, and weight-for-age Z-score at baseline (*p* > 0.05). However, significant between-group differences were observed for BMI, height-for-age Z-score, and BMI-for-age Z-score. Children in the NE-only group had significantly higher baseline BMIs (13.5 ± 0.7 kg/m^2^ vs. 13.0 ± 0.7 kg/m^2^; *p* = 0.001) and BMI-for-age Z-scores (−1.4 ± 0.6 vs. −1.9 ± 0.7; *p* = 0.001) compared with the ONS + NE group. In contrast, the ONS + NE group demonstrated a significantly higher height-for-age Z-score than the NE-only group (−1.9 ± 0.7 vs. −2.2 ± 0.5; *p* = 0.013).

#### 3.2.2. Mean Changes in Anthropometric Profiles at Each Timepoint

**Figure 3:**
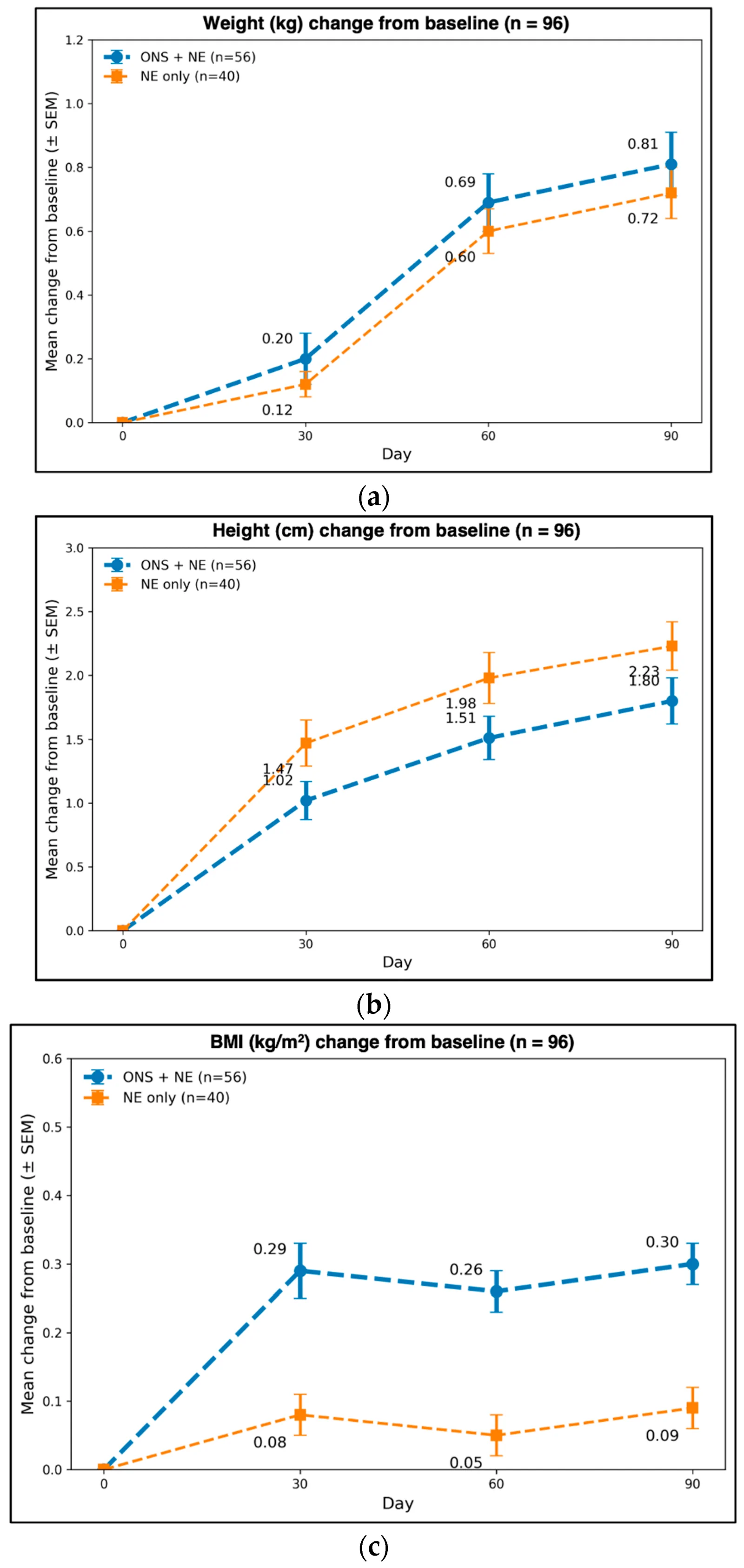
Mean changes in (**a**) weight (kg), (**b**) height (cm), and (**c**) body mass index (BMI; kg/m^2^) from baseline to day 30, day 60, and day 90 among children receiving oral nutritional supplementation with nutrition education (ONS + NE) and nutrition education alone (NE only). Data are presented as the mean ± standard error of the mean (SEM).

**Figure 4:**
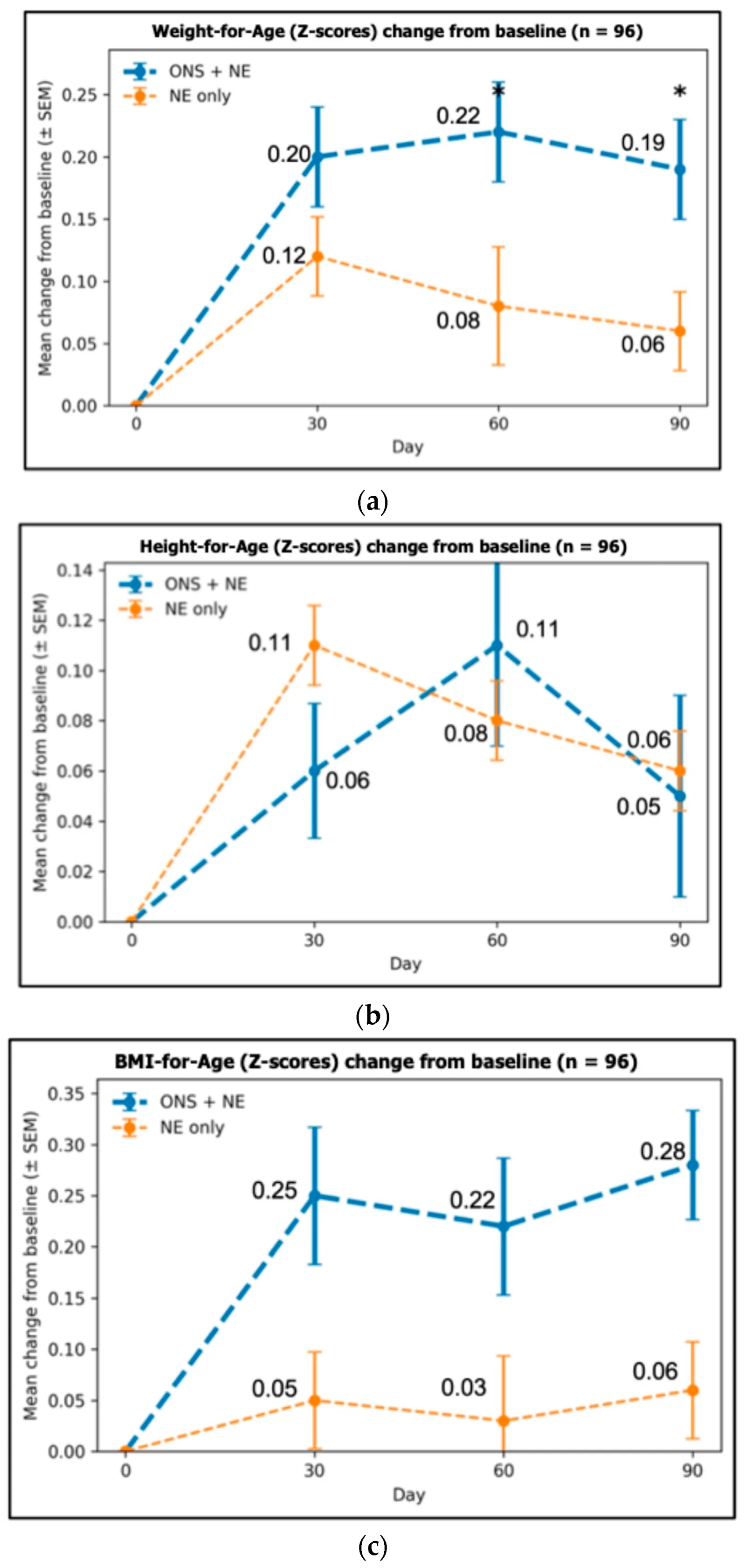
Mean changes in (**a**) weight-for-age Z-score (WAZ), (**b**) height-for-age Z-score (HAZ), and (**c**) BMI-for-age Z-score (BAZ) from baseline to day 30, day 60, and day 90 among children receiving oral nutritional supplementation with nutrition education (ONS + NE) and nutrition education alone (NE-only). Data are presented as the mean ± standard error of the mean (SEM). * Significant between-group differences after adjustment for baseline differences. Detailed adjusted ANCOVA estimates, including adjusted mean differences, 95% confidence intervals, and partial η^2^ effect sizes, are presented in Supplementary Table S1.

Figure 3 shows the mean changes in weight, height, and BMI at baseline, day 30, day 60, and day 90 among children receiving oral nutritional supplementation with nutrition education (ONS + NE) and nutrition education alone (NE). Both groups demonstrated gradual improvements in anthropometric outcomes throughout the intervention period. The ONS + NE group showed slightly greater increases in weight and BMI, whereas the NE group demonstrated slightly greater height gain over time. However, no significant between-group differences were observed across the study period. Figure 4 shows the mean changes in WAZ, HAZ, and BAZ throughout the 90-day intervention period. Both groups demonstrated gradual improvements in weight- and BMI-related z-scores over time. The ONS + NE group consistently showed increases in WAZ and BAZ compared with the NE-only group across all follow-up time points. For HAZ, both groups exhibited small increases over time, with similar trends between groups. After adjustment for baseline values, significant between-group differences were observed only for WAZ at day 60 (adjusted mean difference = 0.140, 95% CI: 0.019–0.261; *p* = 0.024; partial η^2^ = 0.054) and day 90 (adjusted mean difference = 0.122, 95% CI: 0.002–0.243; *p* = 0.047; partial η^2^ = 0.042). Detailed adjusted ANCOVA estimates are presented in Supplementary Table S1.

### 3.3. Secondary Outcomes

#### 3.3.1. 24 h Dietary Recall

Table 4 presents the dietary nutrient intake of children at baseline, day 60, and day 90. At baseline, no significant between-group differences were observed for most nutrients, except thiamin intake, which was significantly lower in the ONS + NE group compared with the NE-only group (0.40 ± 0.13 mg vs. 0.53 ± 0.25 mg; *p* = 0.013). At day 60 and day 90, children in the ONS + NE group demonstrated significantly higher intakes of energy, carbohydrates, protein, fat, potassium, calcium, vitamin A, vitamin C, thiamin, riboflavin, and niacin compared with the NE-only group (all *p* < 0.05). No significant between-group differences were observed for sodium and iron intake throughout the intervention period (all *p* > 0.05). Overall, the findings indicate that oral nutritional supplementation combined with nutrition education improved macro- and micronutrient intake among underweight preschool children compared with nutrition education alone.

Table 5 presents the percentage of catch-up growth energy requirement (ER) achieved among children in the oral nutritional supplementation with nutrition education ONS + NE group and the NE-only group. At baseline, no significant difference was observed between groups in the percentage of catch-up growth ER achieved (*p* = 0.397). However, at day 60 and day 90, the ONS + NE group demonstrated significantly higher percentages of catch-up growth ER achieved compared with the NE-only group (116.6 ± 38.5 vs. 99.1 ± 17.4, *p* = 0.029; and 114.6 ± 37.2 vs. 95.5 ± 23.8, *p* = 0.021, respectively). Similarly, the adjusted mean percentage change from baseline was significantly greater in the ONS + NE group at day 60 (adjusted mean difference = 21.38%, 95% CI: 8.30–34.47; *p* = 0.002; partial η^2^ = 0.156) and day 90 (adjusted mean difference = 24.84%, 95% CI: 11.49–38.18; *p* < 0.001; partial η^2^ = 0.193). Detailed adjusted ANCOVA estimates are presented in Supplementary Table S2. These findings indicate that children receiving ONS combined with nutrition education achieved greater catch-up growth in energy requirements over the intervention period compared with children receiving nutrition education alone.

#### 3.3.2. Appetite and Common Illness

Table 6 presents the effects of the intervention on appetite score and parental reports of common illnesses among children receiving oral nutritional supplementation with nutrition education (ONS + NE) and nutrition education only (NE only). At baseline, no significant difference in appetite score was observed between groups (*p* = 0.455). However, at day 90, children in the ONS + NE group demonstrated a significantly higher appetite score than the NE-only group (4.19 ± 0.64 vs. 3.87 ± 0.57). After adjustment for baseline appetite score and sex, the adjusted mean difference was 0.33 (95% CI: 0.06–0.60; *p* = 0.016), with a moderate intervention effect (partial η^2^ = 0.096). Detailed adjusted ANCOVA estimates are presented in Supplementary Table S2. For reported common illnesses, a higher proportion of children in the ONS + NE group showed improvement compared with the NE-only group (21.9% vs. 3.3%), although the difference did not reach statistical significance (*p* = 0.054).

## 4. Discussion

This multi-centre community-based randomised controlled trial evaluated the effectiveness of oral nutritional supplementation (ONS) combined with nutrition education on growth indicators, dietary intake, appetite, and health outcomes among underweight preschool children in the urban area of Kuala Lumpur. Overall, the findings demonstrated that children receiving ONS together with nutrition education achieved greater improvements in WAZ scores, dietary adequacy, appetite score, and achievement of ER for catch-up growth compared with children receiving nutrition education alone. These findings support the effectiveness of ONS as a nutritional strategy for underweight preschool children living in urban community settings.

At baseline, both intervention groups were generally comparable in sociodemographic characteristics, indicating successful randomisation and minimising the likelihood of major confounding effects related to socioeconomic background and parental characteristics. Nevertheless, significant differences were observed in BMI, BMI-for-age z-score (BAZ), and height-for-age z-score (HAZ) at baseline. Such differences may reflect the heterogeneous nutritional and growth profiles commonly observed among underweight preschool children in community settings, particularly within relatively modest sample sizes. Importantly, baseline-adjusted analyses were conducted to account for these differences, thereby strengthening the validity of the observed intervention effects.

Both intervention groups demonstrated progressive improvements in anthropometric outcomes throughout the 90-day intervention period, suggesting that nutrition education alone may provide some beneficial effects on child feeding practices and dietary behaviours. However, children receiving ONS combined with nutrition education consistently exhibited greater increases in weight-for-age z-score (WAZ) and BMI-for-age z-score (BAZ), with significant between-group differences observed for WAZ at day 60 and day 90 following baseline adjustment. These findings indicate that ONS may have provided additional nutritional benefits compared to nutrition education alone, particularly in supporting weight recovery and catch-up growth among underweight children. The greater improvements in WAZ and BAZ observed in the ONS + NE group were biologically plausible due to the nutrient-dense composition of ONS products, which provide extra energy and protein, as well as essential micronutrients required for anabolic growth processes, and facilitate the growth of new tissue [36,37]. Underweight preschool children were consistently associated with the experience of inadequate dietary energy and protein intake due to poor appetite or selective eating behaviours, limiting their ability to achieve sufficient caloric intake to support catch-up growth [38]. In this context, ONS may potentially bridge these nutritional deficits by increasing total energy and nutrient consumption. Similar findings have been reported in previous intervention studies among undernourished children. The authors of [16,17,18] demonstrated significantly greater improvements in growth outcomes among children receiving ONS supplementation. Collectively, the present findings reinforce existing evidence supporting the role of ONS in promoting short-term weight recovery among nutritionally vulnerable children. The small effect sizes observed for WAZ indicate that, although the magnitude of the intervention effect was modest, it remained sufficient to produce statistically significant improvements over nutrition education alone.

In contrast, no significant between-group differences were observed for height gain or HAZ despite small improvements in both groups over time. This finding is common given that linear growth recovery typically occurs more gradually than weight gain and often requires intervention periods of 6 months or longer before measurable changes become evident [37,39]. Stunting is an indicator of chronic malnutrition, which reflects cumulative nutritional adequacy and is influenced by multiple factors, including chronic nutrient deficiency, recurrent infections, environmental conditions, and endocrine regulation [38,40]. Previous studies have similarly shown that improvements in weight-related indicators led to changes in linear growth during nutritional rehabilitation [17,18]. The relatively short 90-day intervention duration in the present study may therefore have been insufficient to produce clinically meaningful differences in HAZ between groups. Similarly, no significant between-group differences were observed for BMI or BMI-for-age z-score (BAZ). This is possibly because BMI reflects the relationship between body weight and height; modest weight gains over the relatively short intervention period, together with concurrent increases in height in both groups, may have limited the ability to detect significant between-group differences in BMI-related outcomes [37,39].

The findings from the dietary assessment in the present study showed a substantial improvement in dietary adequacy among children receiving ONS. On day 60 and day 90, children in the ONS + NE group demonstrated significantly higher intakes of energy, macronutrients, potassium, calcium, vitamin A, vitamin C, thiamin, riboflavin, and niacin compared with the NE-only group. These findings indicate that ONS effectively improved both macro- and micronutrient intake among underweight preschool children. Importantly, as the dietary assessment captured total dietary intake, including the oral nutritional supplement (ONS), the observed increases in energy and nutrient intakes reflect the additional nutritional contribution of the supplement during the intervention. The observed between-group difference in total energy intake was lower than expected from the prescribed ONS regimen, which may be explained by several factors. First, incomplete compliance played a key role. Mean ONS compliance was 54.4%, with only 46.4% of participants reaching the 75% threshold. Because dietary calculations incorporated actual ONS intake (verified via returned sachets), sub-optimal compliance directly reduced the overall energy differential. Second, participants may have exhibited dietary compensation (a substitution effect), reducing habitual food intake in response to ONS consumption. Lastly, reporting and recall inaccuracies inherent in dietary assessment logs may have contributed to underestimating total energy intake. Despite these factors, the ONS acted as a complementary nutritional source that enhanced overall nutrient adequacy. Similar improvements in nutrient intake have been reported in previous studies evaluating ONS among undernourished children [17,20]. The increased intake of protein and growth-related micronutrients, such as calcium, vitamin A, and B vitamins, may have further supported the finding of significant improvements in WAZ scores observed among the intervention group, due to supporting skeletal growth, cellular metabolism, and immune function [38].

The significantly greater percentage of catch-up growth energy requirement achieved among children receiving ONS further shows the nutritional benefits of the intervention. Notably, children receiving ONS + NE achieved more than 100% of the estimated energy requirements for catch-up growth at both day 60 and day 90, whereas children receiving nutrition education alone remained below the estimated requirement. These findings indicate that ONS effectively provided sufficient dietary energy to meet the estimated energy requirements for catch-up growth. Adequate energy intake is a fundamental prerequisite for catch-up growth, as it supplies the energy surplus required for tissue accretion and normal growth processes [38]. During the preschool years, meeting these increased energy requirements is particularly important because this developmental stage is characterised by rapid physical growth and cognitive development [41]. Failure to achieve adequate energy requirements necessary for catch-up growth during this period may increase the risk of persistent growth faltering, impaired cognitive development, and poorer long-term health outcomes [41,42]. Therefore, interventions that enable children to achieve the recommended energy requirements for catch-up growth may support optimal growth recovery among underweight preschool children.

The present study also demonstrated significantly improved appetite scores among children receiving ONS at day 90. Appetite impairment is commonly associated with undernutrition and may influence inadequate dietary intake and poor growth [18]. The observed improvement in appetite may suggest a possible mechanism underlying the enhanced nutrient intake and growth outcomes observed in the intervention group. Instead, improved nutritional status may have positively influenced appetite regulation and feeding behaviours. A previous study among undernourished children has similarly reported improvements in appetite following ONS supplementation [17]. Although the improvement in common illness outcomes did not reach statistical significance, a greater proportion of children in the ONS + NE group demonstrated improvement compared with the NE-only group. The borderline significance observed may be attributable to the relatively small sample size and short follow-up duration, which may have limited statistical power to detect meaningful differences in illness outcomes. Nevertheless, the observed trend suggests a potential beneficial effect of ONS supplementation on overall child health. A previous study reported a significant positive association between illnesses and oral nutrition supplementation, further reflecting the possible association of its improvement after supplementation [32]. These findings complement the improvements observed in growth, dietary intake, and appetite outcomes, highlighting the broader potential of nutritional interventions in supporting the well-being of underweight preschool children. However, appetite and common illnesses were assessed using a parent-reported appetite rating scale and a parental-reported illness checklist, respectively, which were adapted from previous studies without a formal validation instrument. Therefore, the appetite and common illness findings should be interpreted with caution because the measure may be subject to reporting bias and its psychometric properties have not been established.

An important consideration when interpreting the findings of this present study is the level of compliance achieved in the intervention group. Although only 46.4% of participants achieved the predefined compliance threshold of consuming at least 75% of the prescribed ONS, the intervention still demonstrated significant improvements in weight-for-age z-score, dietary adequacy, catch-up growth energy requirement achievement, and appetite compared with nutrition education alone. The moderate compliance likely reflects the practical challenges of administering daily oral nutritional supplementation in community preschool settings, where factors such as early ONS refusal due to sensory rejection, parental withdrawal, relocation, and incomplete consumption may influence adherence [17]. Consequently, the observed intervention effects may represent conservative estimates of the true efficacy of ONS under optimal adherence conditions. These findings suggest that ONS can provide meaningful nutritional benefits under routine community implementation conditions; however, the magnitude of the observed effects should be interpreted in the context of the compliance achieved during the intervention.

This study possesses several important strengths. To the authors’ knowledge, this is the first multi-centre community-based randomised controlled trial with two study arms evaluating the effectiveness of ONS among underweight preschool children in Malaysia. The study additionally extends previous Malaysian population evidence by comprehensively evaluating anthropometric outcomes, dietary adequacy, parental-reported appetite, and parental-reported common illnesses within real-world urban community settings rather than hospital-based populations. Nevertheless, several limitations should be acknowledged. This study is a non-blinded, open-label study, which may present bias in administering and allocating treatments to each study group and assessing study outcomes. Participant attrition and incomplete dietary data may have reduced statistical precision. Dietary data, appetite, and common illnesses were parent-reported and may therefore be subject to recall bias and social desirability bias. Additionally, the 90-day intervention period may have been insufficient to detect substantial improvements in linear growth and longer-term health outcomes. Future studies with larger sample sizes, longer intervention durations, and objective biomarkers of nutritional and immune status are warranted to further elucidate the long-term effectiveness of ONS among underweight preschool children.

## 5. Conclusions

This multi-centre community-based randomised controlled trial demonstrated that oral nutritional supplementation, when provided alongside nutrition education, significantly improved weight-related growth outcomes, dietary adequacy, and appetite among underweight preschool children in urban Kuala Lumpur. Compared with nutrition education alone, ONS provided additional nutritional benefits that may contribute to improved nutritional recovery during the 90-day intervention period. The overall findings support the use of ONS as an effective community-based strategy for managing undernutrition among preschool children. As the first Malaysian multi-centre randomised controlled trial evaluating ONS among underweight preschool children, this study contributes important local evidence to inform nutrition policies and intervention programmes aimed at reducing childhood undernutrition in the community setting. Future research should explore the sustainability of these benefits over longer intervention and follow-up periods.

## Figures and Tables

**Table 1:** Sociodemographic characteristics of children.

Characteristics	Total (N = 96) *n* (%)	ONS + NE (*n* = 56) *n* (%)	NE Only (*n* = 40) *n* (%)	*p*-Value
Age (years)	5.5 ± 0.6	5.4 ± 0.5	5.5 ± 0.7	0.595
Male, *n* (%)	52 (54.2)	31 (55.4)	21 (52.5)	0.782
Female, *n* (%)	44 (45.8)	25 (44.6)	19 (47.5)	
Birth Status, *n* (%)				
Term	89 (92.7)	52 (92.9)	37 (92.5)	0.622
Pre-term	7 (7.3)	4 (7.1)	3 (7.5)	
Father’s education Level				
Secondary and below	66 (68.8)	38 (67.9)	20 (70.7)	0.823
Tertiary (cert, Diploma, Degree, Master, PhD)	30 (31.3)	18 (32.1)	12 (30.0)	
Father’s employment status				
Government	32 (33.3)	18 (32.1)	14 (34.2)	0.400
Non-Government	57 (59.4)	32 (57.2)	26 (63.4)	
Not working	7 (7.3)	6 (10.7)	1 (2.4)	
Father smoking’s status				
Yes	59 (59.4)	31 (55.4)	25 (64.1)	0.394
No	39 (40.6)	25 (44.6)	14 (35.9)	
Father’s Income				
<RM 2559 (USD 605.8)	49 (51.1)	27 (48.2)	22 (55.0)	0.759
RM 2559–5249 (USD 606–1242.7)	37 (38.5)	23 (41.1)	14 (35.0)	
>RM 5250/USD 1243	10 (10.4)	6 (10.7)	4 (10.0)	
Mother’s education Level				
Secondary and below	53 (55.2)	27 (48.2)	26 (65.0)	0.103
Tertiary (Certificate, Diploma, Degree, Master, PhD)	43 (44.8)	29 (51.8)	14 (35.0)	
Mother’s employment status				
Government	8 (8.3)	5 (8.9)	3 (7.5)	0.691
Non-Government	28 (29.2)	18 (32.3)	10 (25.0)	
Not working	60 (62.5)	33 (58.9)	27 (67.5)	
Mother’s smoking status				
Yes	1 (1.0)	1 (1.8)	0 (0)	0.396
No	96 (99.0)	55 (98.2)	41 (100.0)	
Mother’s Income				
<RM 2559 (USD 605.8)	71 (74.0)	45 (80.4)	26 (65.0)	0.123
RM 2559–5249 (USD 606–1242.7)	21 (21.8)	8 (14.3)	13 (32.5)	
>RM 5250 (USD 1243)	4 (4.2)	3 (5.3)	1 (2.5)	
Parents’ Marital Status				
Married	90 (93.8)	53 (94.6)	37 (92.5)	0.669
Divorced	6 (6.2)	3 (5.4)	3 (7.5)	

Note: Data are presented as mean ± SD or *n* (%). Between-group comparisons for continuous variables were performed using independent-samples *t*-tests, while categorical variables were compared using chi-square tests or Fisher’s exact tests, as appropriate. Statistical significance was set at *p* < 0.05.

**Table 2:** Intervention adherence among participants allocated to the ONS + NE group (*n* = 56).

Variable	ONS + NE (*n* = 56) Mean (%)
Allocated to ONS + NE	56 (100.0)
ONS refusal at day 7	11 (19.6)
Completed day 90 assessment	38 (67.9)
Compliance rate (≥75% of prescribed ONS)	26 (46.4)
Mean percentage of prescribed ONS consumed	54.4 ± 31.2

Note: ONS was prescribed at two servings per day throughout the planned 90-day intervention. The mean percentage of prescribed ONS consumed was calculated as the total number of ONS servings consumed divided by the total number of prescribed ONS servings, expressed as a percentage. The compliance rate was defined as the proportion of participants who consumed ≥75% of the prescribed ONS (equivalent to an average intake of ≥1.5 servings/day) and was calculated by dividing the number of participants meeting this criterion by the total number of participants allocated to the ONS + NE group.

**Table 3:** Baseline anthropometrics of the children (*n* = 96).

Characteristics	Total (*n* = 96)	ONS + NE (*n* = 56)	NE Only (*n* = 40)	*p*-Value
Age (years)	5.5 ± 0.6	5.4 ± 0.5	5.5 ± 0.6	0.595
Male, *n* (%)	52 (54.2)	31 (55.4)	21 (52.5)	0.782
Female, *n* (%)	44 (45.8)	25 (44.6)	19 (47.5)	
Weight (kg)	13.9 ± 1.1	13.9 ± 1.2	14.1 ± 1.1	0.482
Height (cm)	102.7 ± 4.7	103.2 ± 4.6	101.9 ± 4.7	0.190
BMI (kg/m^2^)	13.2 ± 0.7	13.0 ± 0.7	13.5 ± 0.7	0.001
Z-Scores				
Weight-for-age	−2.5 ± 0.4	−2.5 ± 0.4	−2.4 ± 0.4	0.497
Height-for-age	−2.0 ± 0.7	−1.9 ± 0.7	−2.2 ± 0.5	0.013
BMI-for-age	−1.7 ± 0.7	−1.9 ± 0.7	−1.4 ± 0.6	0.001

Note: Data are presented as mean ± SD. Between-group comparisons for continuous variables were performed using independent-samples *t*-tests, while categorical variables were compared using chi-square tests or Fisher’s exact tests, as appropriate. Statistical significance was set at *p* < 0.05.

**Table 4:** Energy and nutrient intakes between groups at baseline, day 60, and day 90 (*n* = 61).

Variable	Timepoint	All (*n* = 61)	ONS + NE (*n* = 32)	NE Only (*n* = 29)	*p*-Value
Energy (kcal)	Baseline	1349.7 ± 336.0	1321.7 ± 345.1	1381.5 ± 327.0	0.491
	Day 60	1338.5 ± 289.4	1650.1 ± 370.8	1386.1 ± 224.3	<0.001
	Day 90	1341.0 ± 319.8	1626.2 ± 373.9	1327.1 ± 268.7	<0.001
Carbohydrate (g)	Baseline	155.3 ± 38.8	152.2 ± 46.4	159.1 ± 29.3	0.493
	Day 60	157.8 ± 34.6	193.7 ± 43.1	166.5 ± 29.2	0.006
	Day 90	160.0 ± 45.6	192.1 ± 44.7	161.8 ± 47.9	0.013
Protein (g)	Baseline	53.5 ± 15.8	52.6 ± 14.9	54.4 ± 16.7	0.664
	Day 60	58.3 ± 56.2	61.4 ± 14.2	51.0 ± 11.5	0.003
	Day 90	48.6 ± 13.7	58.6 ± 14.3	46.7 ± 13.4	0.001
Fat (g)	Baseline	55.2 ± 15.1	53.5 ± 15.2	56.9 ± 14.9	0.385
	Day 60	53.1 ± 14.7	66.2 ± 16.8	54.4 ± 13.0	0.003
	Day 90	54.9 ± 14.8	67.1 ± 17.8	53.6 ± 11.5	<0.001
Sodium (mg)	Baseline	1704.7 ± 614.8	1646.4 ± 596.1	1770.3 ± 632.7	0.434
	Day 60	1624.6 ± 498.5	1748.7 ± 529.4	1641.5 ± 475.3	0.411
	Day 90	1593.6 ± 577.7	1764.8 ± 700.7	1524.7 ± 442.5	0.119
Potassium (mg)	Baseline	850.8 ± 272.7	837.2 ± 276.7	865.3 ± 270.2	0.690
	Day 60	867.3 ± 305.6	1201.9 ± 351.1	858.1 ± 283.6	<0.001
	Day 90	934.9 ± 423.5	1179.4 ± 390.9	942.2 ± 464.7	0.035
Calcium (mg)	Baseline	475.9 ± 203.7	432.6 ± 211.2	526.4 ± 191.7	0.075
	Day 60	465.8 ± 229.0	800.0 ± 271.1	500.0 ± 200.8	<0.001
	Day 90	472.7 ± 216.8	734.3 ± 207.3	494.2 ± 229.1	<0.001
Iron (mg)	Baseline	13.3 ± 4.4	12.6 ± 4.9	14.1 ± 3.8	0.188
	Day 60	13.0 ± 4.2	13.3 ± 4.3	14.1 ± 4.2	0.467
	Day 90	12.7 ± 4.0	13.9 ± 4.3	12.5 ± 3.8	0.173
Vitamin A (µg RE)	Baseline	777.1 ± 360.7	727.7 ± 311.6	830.5 ± 404.8	0.268
	Day 60	742.3 ± 282.6	1201.8 ± 350.0	773.7 ± 250.1	<0.001
	Day 90	819.6 ± 310.0	1252.4 ± 327.1	756.6 ± 314.6	<0.001
Vitamin C (mg)	Baseline	21.7 ± 15.8	21.1 ± 14.5	22.4 ± 17.1	0.748
	Day 60	24.3 ± 17.6	85.1 ± 20.1	30.2 ± 19.7	<0.001
	Day 90	28.4 ± 26.8	79.4 ± 32.0	28.1 ± 27.5	<0.001
Thiamin (mg)	Baseline	0.46 ± 0.21	0.40 ± 0.13	0.53 ± 0.25	0.013
	Day 60	0.46 ± 0.19	1.20 ± 0.30	0.54 ± 0.19	<0.001
	Day 90	0.53 ± 0.34	1.15 ± 0.36	0.55 ± 0.45	<0.001
Riboflavin (mg)	Baseline	1.14 ± 0.41	1.07 ± 0.40	1.22 ± 0.43	0.178
	Day 60	1.13 ± 0.39	1.83 ± 0.47	1.23 ± 0.39	<0.001
	Day 90	1.23 ± 0.50	1.77 ± 0.49	1.32 ± 0.58	<0.001
Niacin (mg)	Baseline	9.48 ± 3.69	9.17 ± 3.41	9.83 ± 3.96	0.484
	Day 60	9.97 ± 3.29	15.84 ± 3.73	10.87 ± 3.39	<0.001
	Day 90	10.23 ± 3.79	15.34 ± 4.03	10.21 ± 4.29	<0.001

Note: Data are presented as the mean ± SD. ONS + NE—oral nutritional supplementation with nutrition education; NE only—nutrition education only. Statistical significance was set at *p* < 0.05.

**Table 5:** Percentage of catch-up growth energy requirement (ER) achieved and changes by study group (*n* = 61).

% Catch-Up Growth ER Achieved at	ONS + NE (*n* = 32)	NE Only (*n* = 29)	*p*-Value
Baseline	93.4 ± 32.9	100.4 ± 30.9	0.397
Day 60	116.6 ± 38.5	99.1 ± 17.4	0.029 *
Day 90	114.6 ± 37.2	95.5 ± 23.8	0.021 *
Mean % change from Baseline to day 60	23.18 ± 33.72	−1.26 ± 24.56	0.002 *
Mean % change from Baseline to day 90	21.24 ± 31.32	−4.91 ± 23.50	<0.001 *

Note: Values are presented as the mean ± SD, * ANCOVA adjusted for baseline and sex. Statistical significance was set at *p* < 0.05. Detailed adjusted ANCOVA estimates are presented in Supplementary Table S2.

**Table 6:** Effects of intervention on appetite and common illness outcomes (*n* = 62).

Outcome	ONS + NE (*n* = 32)	NE Only (*n* = 30)	*p*-Value
Appetite score (Baseline)	3.78 ± 0.75	3.80 ± 0.71	0.455 ^a^
Appetite score (Day 90)	4.19 ± 0.64	3.87 ± 0.57	0.016 ^b^
Common illness			
Improved	7 (21.9)	1 (3.3)	0.054 ^c^
Not improved	25 (78.1)	29 (96.7)	

Note: Appetite presented as the mean ± SD. Common illnesses presented as *n* (%). Common illnesses included fever, flu, diarrhoea, and vomiting. Appetite and common illness data were parent-reported using a structured questionnaire. Statistical tests: ^a^ baseline comparison using independent *t*-tests (*p* = 0.455), ^b^ between-group comparison using analysis of covariance (adjusted for baseline appetite and sex, ^c^ common illness analysed using Fisher’s exact test. Detailed adjusted ANCOVA estimates are presented in Supplementary Table S2.

## Data Availability

The data presented in this study are available from the corresponding author upon reasonable request. The data are not publicly available due to privacy and ethical restrictions involving human participants.

## Supplementary Materials

The following supporting information can be downloaded at https://www.mdpi.com/article/10.3390/nu18162714/s1. Supplementary Table S1: Adjusted mean changes in anthropometric z-scores estimated using ANCOVA. Supplementary Table S2: Adjusted mean changes and adjusted between-group differences in catch-up growth energy requirements and appetite score estimated using ANCOVA.

## Abbreviations

The following abbreviations are used in this manuscript:

BAZ	BMI-for-Age Z-Score
BMI	Body Mass Index
CI	Confidence Interval
EE	Energy Expenditure
EI	Energy Intake
ER	Energy Requirement
HAZ	Height-for-Age Z-Score
IBW	Ideal Body Weight
ITT	Intention-to-Treat
NE	Nutrition Education
NHMS	National Health and Morbidity Survey
ONS	Oral Nutritional Supplementation
ONS + NE	Oral Nutritional Supplementation plus Nutrition Education
PAL	Physical Activity Level
RNI	Recommended Nutrient Intake
SD	Standard Deviation
SEM	Standard Error of the Mean
WAZ	Weight-for-Age Z-Score
WHO	World Health Organization

## Appendix A

**Table A1:** Nutritional composition of the oral nutritional supplement (ONS) per serving (48 g).

Nutrients	Per Serving (48 g)
Energy (kcal)	219
Carbohydrate (g)	27.1
Protein (g)	6.7
Fat (g)	8.8
Sodium (mg)	87
Potassium (mg)	201.6
Calcium (mg)	226
Iron (mg)	0.8
Vitamin A (µg RE)	302
Vitamin C (mg)	40.8
Thiamin (mg)	0.5
Riboflavin (mg)	0.5
Niacin (mg)	4.1
