# Supplementary material for: The Effectiveness of Oral Nutrition Supplementation on Growth Indicators, Dietary Intake, Appetite, and Health Outcomes Among Underweight Preschool Children in Urban Area of Kuala Lumpur, Malaysia: A Multi-Centre Community-Based Randomised Controlled Trial
Source: Nutrients. 2026 Aug 19;18(16):2714. doi: 10.3390/nu18162714 (PMC13516494; doi:10.3390/nu18162714)
Supplement: Supplementary file 1 [file nutrients-18-02714-s001.zip › nutrients-4457018-supplementary.pdf]

Table S1. Adjusted mean changes in anthropometric z-scores estimated using ANCOVA.

Outcome	Time	Adjusted Mean (ONS+NE)	Adjusted Mean (NE)	Adjusted Mean Difference (ONS+NE − NE) (95% CI)	*P*-Value	Partial η²
**WAZ**	Day 30	0.201	0.120	0.082 (−0.027, 0.190)	0.138	0.024
	Day 60	0.215	0.076	**0.140 (0.019, 0.261)**	**0.024**	0.054
	Day 90	0.186	0.064	**0.122 (0.002, 0.243)**	**0.047**	0.042
**HAZ**	Day 30	0.075	0.097	−0.023 (−0.122, 0.076)	0.650	0.002
	Day 60	0.116	0.073	0.043 (−0.053, 0.139)	0.377	0.009
	Day 90	0.056	0.048	0.008 (−0.084, 0.100)	0.864	0.000
**BAZ**	Day 30	0.245	0.105	0.141 (−0.019, 0.300)	0.084	0.032
	Day 60	0.195	0.075	0.120 (−0.059, 0.299)	0.186	0.019
	Day 90	0.218	0.097	0.121 (−0.060, 0.302)	0.188	0.019

Adjusted means and adjusted mean differences were estimated using analysis of covariance (ANCOVA), adjusting for the corresponding baseline anthropometric z-score and sex. Adjusted mean differences are presented as intervention minus control (ONS+NE − NE) with 95% confidence intervals (95% CI). Partial eta squared (partial η^2) is reported as a standardised measure of effect size. According to Cohen's conventional benchmarks, partial η^2 values of 0.01, 0.06, and 0.14 represent small, medium, and large effect sizes, respectively.

Table S2. Adjusted mean changes and adjusted between-group differences in catch-up growth energy requirements and appetite score estimated using ANCOVA.

Outcome	Time point	ONS + NE (Adjusted Mean)	NE only (Adjusted Mean)	Adjusted Mean Difference (ONS+NE − NE) (95% CI)	*P*-Value	Partial η²
Catch-up growth energy requirement (%)	Day 60	20.85	−0.53	**21.38 (8.30, 34.47)**	**0.002**	0.156
Catch-up growth energy requirement (%)	Day 90	19.03	−5.80	**24.84 (11.49, 38.18)**	**<0.001**	0.193
Appetite score	Day 90	4.19	3.86	**0.33 (0.06, 0.60)**	**0.016**	0.096

Adjusted mean differences (ONS+NE − NE) were estimated using ANCOVA after adjustment for baseline values of the respective outcome and sex. Values are presented as adjusted mean differences with 95% confidence intervals (95% CI). Statistical significance was defined as $p < 0.05$. Partial eta squared (partial η^2) is reported as a standardised measure of effect size. According to Cohen's conventional benchmarks, partial η^2 values of 0.01, 0.06, and 0.14 indicate small, medium, and large effect sizes, respectively.
